# A containerised approach for multiform robotic applications

**DOI:** 10.3389/frobt.2024.1358978

**Published:** 2024-04-24

**Authors:** Giuseppe Cotugno, Rafael Afonso Rodrigues, Graham Deacon, Jelizaveta Konstantinova

**Affiliations:** Ocado Technology, Welwyn Garden City, United Kingdom

**Keywords:** robotic architectures, microservices, robot integration, virtualization, robot software design

## Abstract

As the area of robotics achieves promising results, there is an increasing need to scale robotic software architectures towards real-world domains. Traditionally, robotic architectures are integrated using common frameworks, such as ROS. Therefore, systems with a uniform structure are produced, making it difficult to integrate third party contributions. Virtualisation technologies can simplify the problem, but their use is uncommon in robotics and general integration procedures are still missing. This paper proposes and evaluates a containerised approach for designing and integrating multiform robotic architectures. Our approach aims at augmenting preexisting architectures by including third party contributions. The integration complexity and computational performance of our approach is benchmarked on the EU H2020 SecondHands robotic architecture. Results demonstrate that our approach grants simplicity and flexibility of setup when compared to a non-virtualised version. The computational overhead of using our approach is negligible as resources were optimally exploited.

## 1 Introduction

### 1.1 Motivation

**FIGURE 1:**
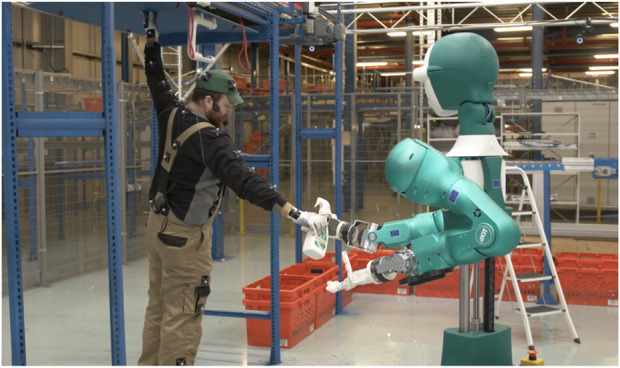
The robotic software architecture developed within the EU H2020 SecondHands project has to enable a humanoid robot to assist a technician during maintenance in real-world conditions. Its several parts constitute a multiform robotic system as they were not developed having a specific robotic framework in mind.

Recently, complete robotic solutions, such as collaborative robots (Asfour et al., 2018) or robotic warehouse automation (Hamberg and Verriet, 2012) are frequently deployed in real world scenarios (Cotugno et al., 2020). For example, the purpose of the EU H2020 SecondHands project^1^ is to develop a humanoid collaborative robot (the ARMAR-6 (Asfour et al., 2018)) to assist a maintenance technician in servicing conveyor belts in a real-world warehouse (Figure 1). The software architecture that is powering such robots can be very complex, with several components interrelated and dependent among each other.

Traditionally, robotic frameworks, like ROS, are used to simplify the development and integration of robotic software architectures. However, relying on a singular robotic framework makes the resulting system uniform as the set of software libraries and development tools, used to develop and interconnect core components instrumental to the robot’s autonomy, is predefined and could be embedded in the wider code base by design or necessity. In addition, every component must be developed with a predefined structure. The expectation from using such robotic frameworks is that components developed by a research group can be easily integrated to a different architecture which runs the same framework.

Integrating third party software is becoming even more critical today, as various contributions valuable for robotics are shared online. For example, in SecondHands, image segmentation is performed using MaskRCNN (He et al., 2017): a deep network developed for segmenting common objects which has been re-trained for detecting tools. Robotic household assistants, competing in the Robot@Home competition, implement speech understanding by integrating several language processing components developed for general use (Matamoros et al., 2018). Such contributions are often developed without following the integration rules imposed by a robotic framework and it can be difficult to include them in a large system. Such contributions are called *multiform* in this paper as they do not conform to the development rules of a robotic framework and are constituted by several heterogeneous components.

For these reasons, our paper proposes a general purpose approach which facilitates the inclusion and interoperation of third party software into an existing robotic architecture. Our approach relies on the use of containers, which are lightweight virtual machines. The contributions of this paper are as follows:

• A container-based methodology is proposed, which allows systematic and minimally invasive integration with an existing robotic framework;
• The use of the containerised approach is demonstrated on a real world application of collaborative robot within the SecondHands project;
• The overall system is evaluated against a non-virtualised version in terms of integration complexity and run-time computational performance.

This paper is asking the following research question: *Is it possible to systematically design or incrementally adapt a robotic system to include and interoperate existing third party components?* In this work, a *third party component* is defined as a contribution developed by different developers not directly involved with the integration of a specific robotic system. The component might not follow any integration rule established for the system, for example it could be written in a different unsupported language, use a different robotic framework or run standalone, use incompatible libraries or different build tools, etc. Such a component can be a prototype proven to work standalone, whose integration in a larger system might be not-trivial. This differs from the scenario where a component has been developed using a robotic framework as that framework imposes rules that must be respected for the component to be useable. It is important to underline that our methodology suggests a set of principles to facilitate the systematic integration of components as opposed to an *ad hoc* approach which integrates third party components differently and might have different outcomes in terms of simplicity of integration and performance cost for different components. In the Evaluation section of the paper we will test two hypotheses: *1. Following all the guidelines of our methodology simplifies the integration complexity* and *2. Following all the guidelines of our methodology has a noticeable performance cost*.

The structure of this paper is as follows: in Section 1.2 we compare our work to the state of the art, while in Section 2 our methodology is described and applied to the SecondHands robot architecture. Section 3 quantifies the integration complexity and runtime performance costs of using our methodology. Finally, Section 4.1 discusses our results and Section 4.2 summarises the key findings and limitations of the paper proposing avenues for future work.

### 1.2 Related work

Over the decades, the number of proprietary and open source robotic frameworks, used for software integration, has increased greatly. The YARP framework (Metta et al., 2006), is a cross-platform framework which mostly targets the humanoid robot iCub (Metta et al., 2008). The well-known ROS framework can interoperate with a large number of robots. Other less known frameworks are also targeted to a specific robot (NaoQi (Pot et al., 2009), Khepera III (Cotugno et al., 2011)), a family of robots (ArmarX (Vahrenkamp et al., 2015)) or a limited predefined selection of robots (OpenRDK (Calisi et al., 2008)). It is beyond the scope of this paper to propose a survey of the features of state-of-the-art robotic frameworks (Mohamed et al., 2008). However, we identify three common features: 1) implementation of a smallest entity able to provide some functionality (e.g. node, module), 2) definition of a communication protocol for exchanging information across those entities, 3) definition of a development methodology and standardised set of development tools. As a result, robotic architectures are deployed as distributed systems and are uniform: components have the same structure.

The homogeneity imposed by a robotic framework becomes a limitation when a robotic architecture includes third party software. Third party contributions have to be adapted to fit the design and tools imposed by the framework itself (Khandelwal et al., 2017). Yet such integrations might prove to be non-trivial (Cervera, 2019) and integrating components from two different frameworks adds complexity (Randazzo et al., 2018) even when the same hardware is used. For example, the SecondHands system integrates off-the-shelf deep networks and components developed differently by five research groups, making it a multiform system. In such system, it is impractical to re-implement every contribution to comply to the rules of a robotic framework. Code re-use can be achieved using virtualisation. In literature, two virtualisation approaches are popular: virtual machines (VMs) and containers. A VM is a software which emulates a PC both in its hardware and operative system. A container is a light-weight high performance (Seo et al., 2014) VM which shares with its host (the PC) only the kernel.

Within robotics, virtualisation has been pioneered by several authors. In Fres et al. (Fres and Alonso, 2010) a virtual machine is used to encapsulate the controller for a mobile robot using a novel programming language, while (Hinze et al., 2018) employs containers to run multiform grasping simulations with real-time control requirements. The limitation of these approaches is that the proposed systems are not designed to support a complex robotic architecture, as virtualisation is not used at its full power. In some cases, virtualisation is used merely as a tool to bypass a certain issue. For example, in (Rodrigues, 2017), containers are used for dynamical deployment of control software on mobile robots via the cloud, optimising the workload as needed. In (Liu et al., 2018), containers are used as a base infrastructure to support the learning and deployment of control strategies to insert pegs into holes. Those works solve a well-defined problem, however they cannot be applied to similar problems. Virtualisation here is used as a tool rather than being a structural part of the architecture.

Other works, instead, use virtualisation as a structural component of a larger framework. For example, Mohanarajah et al. (Mohanarajah et al., 2014) proposes a full robotic framework which relies on containers to execute different robot algorithms, while in (Turnbull and Samanta, 2013), virtual machines are used to provide different services for cloud robotics applications. The shortcoming of such approaches is that it is not described how the proposed frameworks can be integrated with preexisting robotic software without rewriting the old code. Design principles must be provided to guide the development of large multiform robotic architectures. Distributed systems can be used as a source of inspiration.

Modern distributed systems (e.g. cloud video or audio streaming platforms) implement a *microservice architecture* (Newman, 2015), where many heterogeneous components are deployed and networked across several machines to contain faults and balance high computational loads. A microservice-inspired framework has been already pioneered in (Wang et al., 2019) for coordinating and preparing robot software for deployment. In this work the use of ROS is compulsory, in contrast with our approach which is robotic framework-agnostic.

In order to develop reusable components, that can be deployed on different robotic architectures, there is a need to shift designs from monolithc architectures to more modular microservice-like architecture. Our work establishes a proposed approach to encode such modularity by design.

## 2 Methodology

The aim of the proposed containerised approach is to define design principles to facilitate the construction of a robotic multiform modular architecture whose elements can be integrated in a preexisting robotic system. The approach is based on three fundamental principles derived from microservice architectures: componentisation, virtualisation and automation (Newman, 2015). Table 1 summarises its theoretical foundations. Our approach favours the reuse of preexisting code, but the approach can be used as guidelines for a new robotic framework as well. Developing a new framework from the beginning might sound ideal but it is not always possible or feasible, especially in industrial applications. In industry it might be more prudent to refactor existing code bases, proven to work in a real world production setting, and to incrementally evolve a robotic system to serve ever expanding requirements. With this assumption, rewriting a framework is costly and has high risks due to the fact that bugs can be introduced at any time and it takes time and effort to bring a new framework to feature parity with a previous code base. Additionally, an initial well thought design might prove to be limited by the time it is deployed to production as requirements might have changed in the meantime. As such, we conceived our approach for adapting existing code as this is a more frequent scenario than a full redesign.

**TABLE 1:** Founding principles of the proposed containerised approach for multiform robotic architectures.

Principle	Explanation	Properties	Properties description
A. Componentisation	Dividing complex robot software into well defined software units	1. Defined Functionalities	Identification of operations that a component provides to a client. The definition of a component is specific for the target application
		2. Tested in Isolation	Must always be possible to execute a component in isolation, using mock-up inputs/outputs if needed, to simplify testing
		3. Defined Interfaces	Clear means to interact with a component to execute its operations
B. Virtualisation	Creating a virtual environment where a component can operate	1. Defined Working Environment	Construction of a virtual environment easy to execute where a component operates, ideally fully isolated from the host
		2. Cloud Hosting	Individual virtualised components, tested to be executable, must be accessible by users from the cloud
		3. Subsystem Preparation	Optionally, an ensemble of virtualised components can be automatically fetched from the cloud and assembled in a subsystem working out of the box
C. Automation	Automatically preparing, testing and sharing virtualised components on the cloud	1. Automatic Build of Blueprints	Blueprints (templates) of the components and their virtual environment must be automatically prepared for every improvement
		2. Automatic Testing of Blueprints	New developments of a virtualised component shall be automatically tested to ensure that the component can at a minimum be executed with no errors
		3. Automatic Updates on the Cloud	Updated and tested blueprints of virtualised components should be automatically shared on the cloud to be retrieved by a third party
		4. Automatic Versioning	Blueprints and components’ code must be automatically versioned and kept in sync, so that specific versions can be obtained predictably

Our approach relies on the creation of blueprints: virtual environments adapted for the execution of components. To use a virtualised component a container is generated from the blueprint, which acts as a template. We chose docker^2^ for creating blueprints as it is an industry standard and it has tools for automatically deploying subsystems from the cloud (i.e. *docker-compose*). Additionally, several operations of our methodology (Table 1. *C*) are automated using a Continuous Integration (CI) tool, which is a tool designed for automating software development tasks. We chose Travis CI for this as it is a cloud-based CI, but others are also suitable.

**FIGURE 2:**
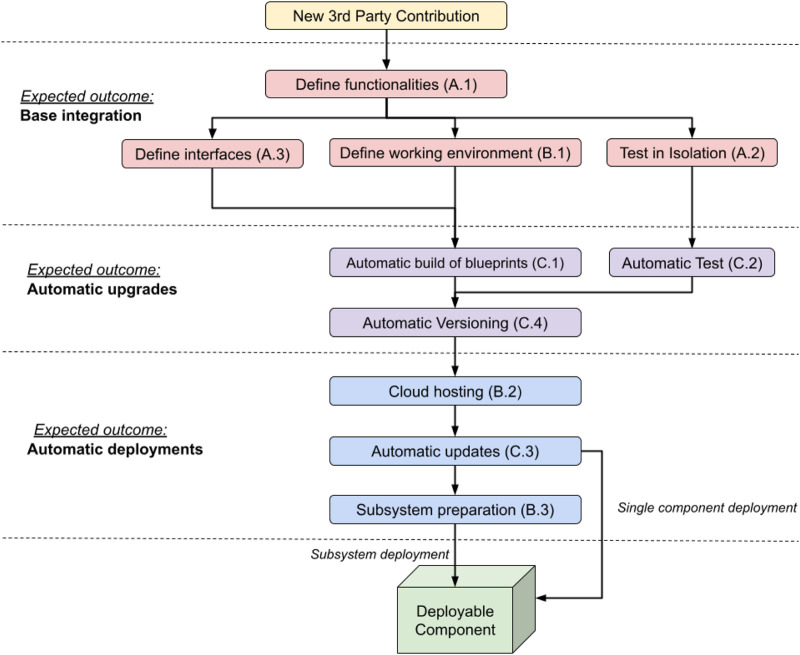
Proposed workflow of our methodology. Properties are grouped based on the expected outcome obtained by their application. Arrows indicate dependencies, e.g. cloud hosting should not be implemented if versioned blueprints are not available. Properties that are at the same level can be implemented concurrently. Please note that Principle B.3 is optional and its application is recommended if components are to be deployed together as a subsystem. The workflow can be applied to a new contribution or a pre-existent codebase, in which case A.1 can be used to decide features to factor out into an isolated component to be handled separately.

According to our methodology, for the integration of a heterogeneous component it is required to follow the below workflow, summarised in Figure 2:

1. Define which operations the component will perform. Evaluate the opportunity of factoring out existing features integrated in a pre-existing robotic framework, if it is appropriate to do so at this stage (Table 1: *A.1*).
2.1. Define how the new component will communicate with the existing code base and other components (Table 1: *A.3*).
2.2. Create a blueprint for the heterogeneous component using docker, making sure that the component can be executed correctly (Table 1: *B.1*).
2.3. Add the chosen communication interfaces to the blueprint and test it in isolation. In our case, those were interface definitions in the ArmarX native communication library, but other approaches like e.g. ROS service handlers can be used for different systems. Other components and input required for testing can be included in the blueprint as a mock-up (Table 1: *A.2*).
3. Configure a CI, in our case Travis CI, to automatically prepare and test in isolation the blueprint at every commit (Table 1: *C.1*, *C.2*).
4. Ensure components are versioned appropriately by Travis CI (Table 1: *C.4*).
5. Once testing is completed, the blueprint is uploaded on the cloud (e.g. Google cloud in our case) and new versions are automatically uploaded by Travis CI (Table 1: *B.2*, *C.3*).
6. Optionally, a script can be prepared to automatically fetch and interconnect all the components of a subsystem of the robot (Table 1: *B.3*) using *docker-compose.* This facilitates the deployment of components closely related to each other.

Our methodology is an industry perspective on how to integrate multiform components to produce robotic systems ready for a production scenario. It builds up from principles well assessed in microservice development and applies them to robotics. Our contribution suggests an approach to make integration systematic and less error prone, which are key requirements in industry to ensure that robotic systems are reliable and robust from their first production release. We did find that poor integration can negatively impact the performance of novel research contributions to the point that they cannot be used in a production setting (Triantafyllou et al., 2021). Our approach is demonstrated on the real-world scenario of the SecondHands project^3^. The SecondHands robot (the ARMAR-6 (Asfour et al., 2018)) has a mobile base, multimodal sensory capabilities and the ability to physically and verbally interact with humans. Using its sensors, the robot has to predict and provide the help adequate to the situation. Our methodology can be applied to other architectures as it is not mandatory to refactor pre-existing code encapsulated into a robotic framework, such as ROS, if this is undesirable. Such code can be treated as a stand alone component, with well defined interfaces and responsibilities. Those responsibilities can be as broad as practically feasible to ensure the right balance between timely and incremental deliveries of new functionalities and overall architectural cohesion. Also, it is always possible to refactor any component, or to wrap them up with necessary boilerplate code, in order to fulfil the Properties of *Principle A*, if such a component does not adhere to them already. The Properties of *Principle B* can be applied to any working software as, to the best of the authors’ knowledge, it is unlikely that a software cannot run in a virtual environment sharable over the cloud. The Properties of *Principle C* are best practices to follow to guarantee consistency, mostly enforced with robust CI pipelines. Also in this case, to the best of authors’ knowledge, there is no reason to assume that CI pipelines cannot be crafted to fulfil the above Properties. Since our methodology does not require a mandatory rewrite of previous software, even if integrated in a robotic framework, and software can be reshaped to fulfil the Properties of our methodology, we believe that our approach can be generalised to other architectures.

**FIGURE 3:**
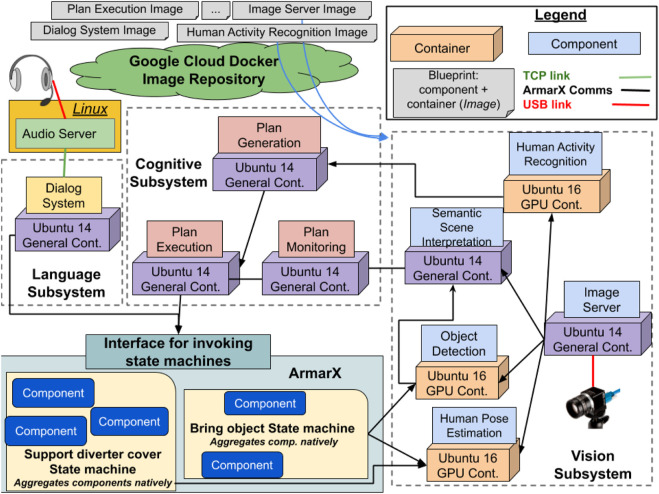
Overview of the SecondHands architecture. Blueprints (templates) of components and subsystems are available on Google Cloud. In the original architecture, a subsystem can be deployed on a dedicated PC. The multiform part of the system (cuboids and squares) only calls ArmarX’s state machines using a dedicated interface. ArmarX can call the multiform components directly. All communication are handled using ArmarX native communication libraries.

The SecondHands architecture, shown in Figure 3, is augmenting the ArmarX robotics framework (Vahrenkamp et al., 2015) which provides several base functionalities and it is the framework used to operate the robot. The uniform structure of ArmarX cannot be altered as it is used for several other applications. All the other components of SecondHands have been developed by different research groups independently from ArmarX and represent the multiform part of the system. Such components have been integrated in a virtual environment and networked using ArmarX’s native communication libraries. This was a design choice aimed at maximising compatibility with ArmarX. Components and their own virtual environments were made available from Google Cloud in the form of blueprints. Within SecondHands, two base blueprints were developed: one providing GPU support (for, e.g. neural networks) and another one more lightweight and without GPU support. For every change, blueprints were automatically built and tested in isolation by Travis CI, using mock-up components and inputs when required. A successfully built and tested blueprint was automatically uploaded on the cloud and versioned by Travis, following the four Properties of Principle C. The multiform part of the system communicates with ArmarX by requesting the execution of a state machine (Vahrenkamp et al., 2015) to perform a particular task, like handing over a tool. An ArmarX state machine relies on several ArmarX components and can request output from an heterogeneous component, such as the detection of the posture of the technician. This design choice allows to handle ArmarX itself as a component with Defined Interfaces and Functionalities (Table 1: *A.1*, *A.3*) whose internal changes do not affect the rest of the architecture. Similarly, internal changes to other heterogeneous components do not affect the rest of the system as components are isolated in their own containers. An exhaustive description of the functionalities of the SecondHands architecture is beyond the scope of this section, Section 3 describes its most important parts. As a result, our methodology extended ArmarX with several new components originally developed in different ways. The components are grouped in three subsystems: Cognitive, Language and Vision, which can be fetched and interconnected automatically using *docker-compose* scripts. The SecondHands robot is equipped with four on-board PCs with different hardware available. A detailed overview of the available hardware is given in Section 3.1. Figure 3 shows how every component is deployed on the robot for its usual operations, where every subsystem, including ArmarX itself, is deployed on a PC. Components are deployed on each machine leveraging *Properties B.3 and B.2* (Subsystem Preparation and Cloud Hosting), as every machine has a *docker-compose* configuration used to bring up the latest stable versions of every container, downloading them from the cloud if required. By using an appropriate versioning system (Principle C.4) it is possible to guarantee that only components tested and confirmed to work together will be brought up.

## 3 Evaluation

In this section, the SecondHands architecture, presented in Section 3.1 is used to evaluate the impact on resources overhead and integration complexity when all the principles of our methodology are followed and when they are not. The aim of the evaluation is to verify hypotheses 1 and 2, assessing if our methodology simplifies the complexity of integration and what is the added performance cost of using it. The evaluation criteria for the integration complexity are the number and type of modifications to a PC’s configuration required to install a given component. The evaluation criterion for the resource overhead is the additional load placed by docker containers on the PC’s resources (RAM, CPU, GPU and network traffic) when compared with the same system running natively.

### 3.1 Experimental setup

A scaled down version of the SecondHands architecture of Section 2, integrated using our approach (*Docker setup*), is compared to a *Native setup* of the same system which did not follow our approach. Both systems are expected to recognise the actions and speech of a technician working in a realistic environment and to control robot’s hardware. The components of the evaluated system are: 1) the *Human Activity Recognition Component* (Alati et al., 2019) which uses a neural network running on a GPU to detect the technician’s actions, 2) an *Image Server* which broadcasts images from a camera, 3) a *Dialogue System Component* (Constantin et al., 2018) which processes natural language, and 4) other components which form the infrastructure of the system (e.g. interfaces with ArmarX, communication among components, etc.).

The components were deployed on a replica of the computational infrastructure of the ARMAR-6 in Ocado Technology’s Robotics Research lab. The infrastructure consists of four workstations, all Quad-core i7 Pentiums with 16 GB RAM, running Ubuntu 14, networked over Ethernet via a switch and connected to the ARMAR-6 hand, cameras and sound system. The four workstations are identified with a name describing their role: *1*) The *Vision PC* has an Nvidia GeForce GTX 1080 GPU to support vision processing and is directly connected to the robot’s cameras (i.e. PrimenSense Carmine). *2*) The *Speech PC* is connected to microphones and a PreSonus AudioBox iTwo sound system to support natural language processing. *3*) The *Real-Time PC* has interfaces to the robot’s hardware and controls an ARMAR-6 humanoid hand (Asfour et al., 2018). The hand is underactuated, has two degrees of freedom and can be operated only via ArmarX. It is operated to demonstrate that the integration of all the components is functional even for complex robotic hardware. *4*) The *Planning PC* is used for any other remaining functionality. In our setup, the communication was administrated by the *Real-Time PC* to further increase bandwidth consumption and stress the system.

### 3.2 Results: integration complexity

The integration complexity evaluation assesses how our approach influences the ease of integration of a multiform system in the worst case scenario. The complexity is measured in terms of additional configurations of the PC required to integrate and execute a component. To evaluate the integration complexity in its worst case scenario, the hard constraint of not altering the original component’s code or container’s blueprint was imposed. The evaluation is performed on a fully integrated system, so that interactions can be captured in a realistic setup. Additionally, for the Native setup, components were deployed on the least possible number of machines to maximise the interactions. Similarly, for the Docker setup, components were as isolated as possible from the host (the PC) as this setup is more complex. As such, it was attempted to deploy both setups only on the *Vision PC*, accessing the sound system on the *Speech PC* remotely, using Linux’s audio server (Pulseaudio).

We classified sources of complexity in four main categories: 1) unset environment variables, 2) missing host configurations, 3) library incompatibilities, 4) driver incompatibilities. The first two categories are easy to address, requiring either to run a setup script or a persistent modification of the PC’s configuration files (for, e.g. driver loading). However, they still require prior knowledge, i.e. a documented procedure. The last two categories are more complex to handle. Library incompatibilities appear when two components require conflicting versions of a library. If solvable, such issues require an *ad hoc* workaround. Driver incompatibilities are when the Linux kernel does not support a driver and a new Linux version needs to be installed on the PC.

When integrating a Native setup, it was observed that most components required a setup script to configure the working environment, while the Dialogue component required a customised host configuration. The most serious issue found was a GPU driver incompatibility with the Activity Recognition component that can only be solved by upgrading the operating system. As such the *Vision PC* was upgraded and the other components were deployed on the *Planning PC*. The camera was also relocated to the *Planning PC* since, to the best of our knowledge, it cannot be accessed remotely. The audio system on the *Speech PC* was still remotely accessed through Pulseaudio. The *Real-Time PC* was left unchanged. When integrating the Docker setup, no issues were experienced besides configuring the containers’ networking. As such, to allow for a comparison with the Native setup, the Docker setup was deployed on the same machines. It is still possible to deploy the full system on a single machine if our methodology is fully followed. Hence, a third containerised setup (*All-in-one setup*), where all components co-exist on the *Vision PC*, was prepared. This setup was also evaluated in Section 3.3. The purpose of this additional evaluation is to give more comprehensive results for testing hypothesis 2, considering also the case where a single machine is bearing the load of the full system as there is no technical limitation preventing this to happen.

It can be observed that the Docker setup was easier to prepare as component’s blueprints were ready to be used. Using blueprints, prepared as indicated in our approach, the setup is delegated to the original developers, who know their components more thoroughly than end-users and can pre-configure them easily.

### 3.3 Results: workload analysis

**FIGURE 4:**
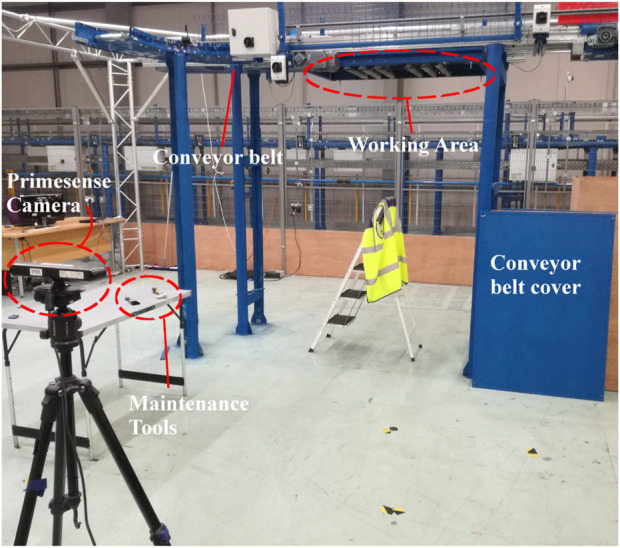
Experimental setup closely representing a real warehouse. A human operator had to wear a high visibility vest and perform maintenance in the working area using real tools.

Workload analysis is performed to understand the computational costs of employing our approach. The three setups produced in Section 3.2, Docker, Native and All-in-one, processed a live simplified maintenance sequence performed in a close reproduction of a real warehouse, shown in Figure 4. The sequence required a human operator to dismantle a conveyor belt’s protective cover causing the ARMAR-6 hand to close. Afterwards he would fetch and climb on a ladder to ask for a brush, clean the conveyor, extend the arm to give away the brush and give a verbal “stop” command, causing the ARMAR-6 hand to open. An example sequence can be viewed in the Supplementary Material. The sequence was performed 16 times by two people for a total of 48 experiments. Any trial which failed to operate the hand was repeated. The three setups were all assessed in terms of network bandwidth, memory, CPU and GPU workloads.

As described in Section 3.2, the Docker and Native setups were deployed on two PCs, while the All-in-one setup was deployed on the GPU-equipped *Vision PC*. For the Native setup, the CPU and RAM usages were monitored using the python system profiling library, *psutil*, while the Network bandwidth was monitored with the Linux’s network traffic monitoring utility, *nethogs*. Only processes related to each component of the system were monitored via *psutil*. For the Docker and All-in-one setups, the same information was accessible through *docker stats* ^4^, docker’s native monitoring tools, which profile CPU, RAM and network bandwidth and are the industry standard tools used to evaluate containers’ key performance metrics. *docker stats* require the docker daemon to run. The daemon is also required to run containers. The computational cost of running the daemon is fixed and does not vary over time, as it is the case for the other components. As such, it was tracked in our results. The daemon is highly optimised to run with minimal footprint in production environments with several containers and the cost of running docker was evaluated to be an additional *0.25%* load on the RAM and less than *0.001%* load on the CPU. GPU workload and memory were measured via Nvidia logging tools for all setups. Only the steady-state part of a trial was analysed. This corresponds to the moment when the first image is broadcast until the hand opens fully. The start-up phase was not considered as the resource consumption is not stable initially and it is not representative of the true load of the system experienced when it is actively used or put under stress during normal operations. In a real world scenario, it is possible to define a process that prevents the system from being used while booting. Readings from individual components were collected at intervals of 250 ms each, relative clock drift measured among computers was negligible.

**FIGURE 5:**
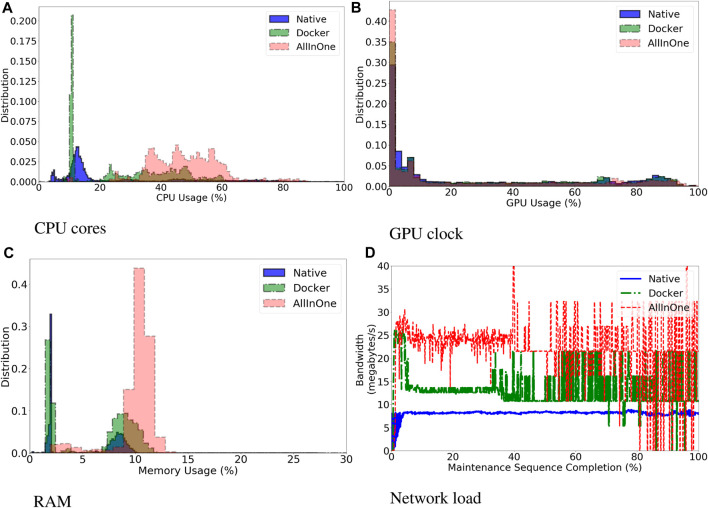
Overall histogram distribution of resource usage, discriminated by system setup, for CPU **(A)**, GPU **(B)** and RAM **(C)**. Plotting number of occurrences of a given load % during the steady-state of the task. Histograms more shifted to the right indicate a more loaded system. Figure **(D)** shows the network load in terms of mean bandwidth consumption over the % of completion of the steady-state of the task. Plotted as a time series.

Figure 5 shows the distribution of resource consumption, expressed in % of total resources used for RAM and GPU, and absolute % of cores used for CPU. Each plot aggregates the data of all trials and components for each setup. Figures 5A, B, C are histograms showing the distribution of resource workload. The *X* axis indicates different workloads while the *Y* axis indicates number of occurrences. A distribution shifted to the right indicates an overall more loaded system. A spike indicates a workload that occurred more often than the others. Figure 5D is a time series, where on the *X* axis is shown the percentage of completion of the maintenance sequence, the steady state of the system, and on the *Y* axis the amount of bandwidth consumed. A summary of the data is shown in Table 2. The GPU memory usage is not reported as it is identical across the three setups. The overall load for each resource was calculated as usage difference relative to the Docker setup (*UD*_*d*_). It was calculated for each resource as follows:

$$\begin{array}{ll} L\left[R_{v}\right] & =\frac{E\left[R_{v}\right]}{C}\\ UD_{d}\left[R\right] & =\frac{L\left[R_{n}\right]-L\left[R_{d}\right]}{L\left[R_{d}\right]} \end{array}$$

Where *v* stands for the setup type (Docker *d*, or Native *n*), *E* [*R*_*v*_] is the mean usage value of a resource *R* (CPU, RAM, GPU) for the setup *v*, *C* is the maximum capacity of a resource, *L* [*R*_*v*_] is the total load percentage of a resource *R* for setup *v*, and *UD*_*d*_ [*R*] is the usage difference for resource *R* relative to the Docker setup (*d*).

**TABLE 2:** Quantitative characterisation of resource consumption. For each resource and setup is shown the mean value and the InterQuartile Range (IQR). Values are percentage of total resource used, for GPU and RAM, and absolute % of CPU cores used.

	Native		Docker		All-in-one	
Resource	Mean	IQR	Mean	IQR	Mean	IQR
CPU	25.72	27.26	29.90	32.85	48.15	16.51
GPU	28.50	57.00	27.86	56.98	24.33	48.81
RAM	5.30	6.52	6.16	7.51	10.07	1.13

The resource usage difference (*UD*_*d*_) is either small (**0.002*pp*** - CPU), slightly more (**+0.43*pp*** - RAM) or slightly less (**−0.60*pp*** - GPU clock). Additionally, as can be observed in Table 2, the differences of the InterQuartile Ranges between Docker and Native setups are 5.59*pp* (CPU), 0.99*pp* (RAM) and −0.02*pp* (GPU clock), which is also small. It can be concluded that the overhead of employing a containerised approach when the system is running at run-time is negligible as the Docker setup does not sensibly affect the overall system load.

The impact of deploying the whole system on one PC was also analysed. The All-in-one setup takes more time to fully load the GPU’s memory (13.20 ± 0.63*s*) when compared to the other two setups (7.91 ± 0.13*s* Native, 10.09 ± 0.25*s* Docker). By observing the histograms of Figure 5, it can be seen that the All-in-one setup has a larger overhead since it is consistently using CPU and RAM more than the other two setups. This can be observed as the distributions on Figures 5A, C are shifted more to the right when compared to the other setups. The reason for such overhead can be seen in Figures 5D. The All-in-one setup produces a higher volume of information at higher speed than the other two setups suggesting that system is transferring more information and, as such, is loading its resources more.

## 4 Discussion and summary

### 4.1 Discussion

The results of Section 3.3 are used to test hypothesis 2: *Following all the guidelines of our methodology has a noticeable performance cost.* The Docker and All-in-one setup follow both all the three principles of the methodology, while the Native setup follows only *Principle A*, since it does use the same components’ code as the other setups. The results demonstrate that the All-in-one setup have a higher workload, as shown by the histograms of Figure 5A higher production of information at a higher speed as seen in Figures 5D. *docker stats* monitors the information at container level, as such a higher bandwidth consumption indicates that the containers communicate more among each other. Those two results combined suggest that, qualitatively, the overall system is able to process more information at the cost of loading the host machine more. A possible explanation of this result is that data transits internally in the operative system via the docker daemon and can be delivered at a faster pace to the recipient.

Indeed, a similar, less pronounced, result can be observed on the Docker setup. In this case, there were more instances when the CPU was less loaded than in the All-in-one setup, as shown by the spike in Figure 5A, although the CPU was still active as its workload was spread between 20% and 60% for the whole steady-state of the trials. This likely happened because the data would have had to be packaged in a format suitable to be transmitted over the physical wire and then unpacked several times as individual packages were sent up over the network until reaching the recipient. This is one of the reasons why communication over a physical wire introduces a lag, which can be exacerbated by network traffic further loading the bandwidth forcing access points to limit the maximum amount of information that can be transferred at the same time. We do believe that in our case, packing and unpacking data over the network stack limited the amount of information travelling and reduced the overall load of every machine, which could process information at a slower pace.

In the All-in-one setup the host machine had to increase its workload to keep up with the information flow as it is able to process more data as soon as this is readily available. In the Docker setup, the host machines were not as loaded. This suggests that the machines could have processed more information if that was available. As such, the Docker setup might be more limited in the amount of information that could process at the same time as it will have to wait for the network stack packing and unpacking data packets before processing them. Another point to underline is that the technical specifications of the host machine for the All-in-one setup were sufficient to handle the full load of the system and to process information at a faster rate. This might not have been possible for a different robotic system, where the overall load could have been excessive for a single machine causing lack of responsiveness or even safety concerns.

This observation suggests there could be benefits in deploying more components on the same host machine as this could save time otherwise spent networking several hosts together. At the same time, overloading a machine could have side effects in the overall responsiveness of the system. Deciding how many hosts to use in a robotic system is a design decision that can be approached quantitatively by evaluating present and expected resource consumption and can keep financial and engineering costs lower. This decision has to be weighed by the fact that the control part of a robot is a real-time system, where timely responsiveness is one of the main factors to consider to ensure the overall safety of the robots and its users.

The results observed for the Docker setup were different from those observed for the Native setup, where the Network load was much lower despite both setups running the same code. This is confirmed by the fact that there were more instances when the CPU was less used than the Docker setup as its distribution in Figure 5A is more shifted to the left. We do believe that our results do not take into account issues with low level configurations of the network stack of the operative system which had an impact on the obtained results. This highlights the benefits of using a containerised approach such as the one presented in our work as docker optimised the network stack for efficient communication without the need to customise the operative system manually. As such, we can conclude that following all the principles of our methodology has a cost which can be offset by a better integration overall, which seems to falsify hypothesis 2 in our scenario.

Our results in Section 3.2 are used to test hypothesis 1: *Following all the guidelines of our methodology simplifies the integration complexity.* The results suggest that having components prepackaged into well defined software units (*Principle A* of our methodology) is a prerequisite but it is not the only aspect to consider. For all three setups the components were prepared according to *Principle A* and yet incompatibilities among components required *ad hoc* adjustments to have an integrated system. *Principle B*, having a virtual environment where the component can operate, was a key factor in facilitating the integration as this allowed to prepare the system in a systematic and streamlined way, identical for every component. *Principle C*, automatically preparing, testing and sharing virtualised components in the cloud, in our case, ensured consistency within components, further consolidating the integration approach in predefined and known steps. It could be that following just *Principles A and B* might be enough to obtain a painless integration. However it might be not sufficient to avoid *ad hoc* configurations and a streamlined integration process due to lack of consistency across components.

### 4.2 Summary

In this paper we proposed a containerised approach, inspired by microservice architectures, and its main principles aimed at augmenting existing robotic architectures with third party components. The principles can also be used as design guidelines for novel robotic frameworks. We applied our methodology to the SecondHands robot architecture and we evaluated our approach, both in terms of integration complexity and computational overhead, against the same architecture deployed without following our approach.

This study demonstrates that our approach grants more flexibility of integration as the same system can be deployed in different ways, even on the same PC, without substantially altering the configuration of the hosting PC. Additionally, we found that containers do not substantially impact the runtime performance of a system. Containerised setups are more reactive than native setups, and systems deployed on a single machine offer the highest reactivity at the cost of a larger workload. Our approach is relevant for robotics as it demonstrates how it is possible to augment an existing system with otherwise incompatible components, limiting the impact on existing code.

Moreover applications designed to run natively come with some form of configuration procedure. Although our approach aims at eliminating the need of any configuration other than deployment, it would be useful to test our approach on other architectures. Additionally, a further analysis of the latency of communication could provide information on the reactivity of native or containerised systems. For the best of our knowledge, it is not possible to evaluate the latency without modifying the original software and, as such, this evaluation will be performed in future work.

Finally, it is worth to note that the key to reusability lies on the quality of the original blueprints. This paper aims at providing guidelines to encourage usability and facilitate integration, however if the blueprints are not designed to be reusable, it is harder to intergate fully modular multiform robotic architectures.

## Data Availability

The raw data supporting the conclusion of this article will be made available by the authors, without undue reservation.

## Footnotes

1 SecondHands: A Robot Assistant For Industrial Maintenance Task, Website: https://secondhands.eu/

2 Docker Community Edition - https://www.docker.com/products/ docker-desktop

3 SecondHands: A Robot Assistant For Industrial Maintenance Task, Website: https://secondhands.eu/

4
*Docker stats* documentation - https://docs.docker.com/engine/ reference/commandline/container_stats/

## References

[B1] Alati E. Mauro L. Ntouskos V. Pirri F. (2019). “Anticipating next goal for robot plan prediction,” in Proceedings of SAI intelligent systems conference (Springer), 792–809.

[B2] Asfour T. Kaul L. Wächter M. Ottenhaus S. Weiner P. Rader S. (2018). “Armar-6: a collaborative humanoid robot for industrial environments,” in 2018 IEEE-RAS 18th international conference on humanoid robots (humanoids) (IEEE), 447–454.

[B3] Calisi D. Censi A. Iocchi L. Nardi D. (2008). “Openrdk: a modular framework for robotic software development,” in 2008 IEEE/RSJ international conference on intelligent robots and systems (IEEE), 1872–1877.

[B4] Cervera E. (2019). Try to start it! the challenge of reusing code in robotics research. IEEE Robotics Automation Lett. 4, 49–56. 10.1109/lra.2018.2878604

[B5] Constantin S. Niehues J. Waibel A. H. (2018). Multi-task learning to improve natural language understanding. *ArXiv* abs/1812.06876.

[B6] Cotugno G. D’Alfonso L. Muraca P. Pugliese P. (2011). “A new extended kalman filter based on actual local information for mobile robots,” in 9th European workshop on advanced control and diagnosis, ACD 2011.

[B7] Cotugno G. Turchi D. Russell D. Deacon G. (2020). “Secondhands: a collaborative maintenance robot for automated warehouses. implications for the industry and the workforce,” in Inclusive robotics for a better society (Springer International Publishing), 195–200.

[B8] Fres O. A. Alonso I. G. (2010). “Rovim: a generic and extensible virtual machine for mobile robots,” in 2010 fifth international conference on systems (IEEE), 37–40.

[B9] Hamberg R. Verriet J. (2012). Automation in warehouse development. Springer.

[B10] He K. Gkioxari G. Dollar P. Girshick R. (2017). “Mask r-cnn,” in Proceedings of the IEEE international conference on computer vision, 2961–2969.

[B11] Hinze C. Tasci T. Lechler A. Verl A. (2018). “Towards real-time capable simulations with a containerized simulation environment,” in 2018 25th international conference on mechatronics and machine vision in practice (M2VIP), 1–6.

[B12] Khandelwal P. Zhang S. Sinapov J. Leonetti M. Thomason J. Yang F. (2017). Bwibots: a platform for bridging the gap between ai and human–robot interaction research. Int. J. Robotics Res. 36, 635–659. 10.1177/0278364916688949

[B13] Liu N. Liu Z. Wei Q. Cui L. (2018). “A containerized simulation platform for robot learning peg-in-hole task,” in 2018 13th IEEE conference on industrial electronics and applications (ICIEA), 1290–1295.

[B14] Matamoros M. Harbusch K. Paulus D. (2018). “From commands to goal-based dialogs: a roadmap to achieve natural language interaction in robocup@ home,” in Robot world cup (Springer), 217–229.

[B15] Metta G. Fitzpatrick P. Natale L. (2006). Yarp: yet another robot platform. Int. J. Adv. Robotic Syst. 3, 8. 10.5772/5761

[B16] Metta G. Sandini G. Vernon D. Natale L. Nori F. (2008). “The icub humanoid robot: an open platform for research in embodied cognition,” in *Proceedings of the 8th workshop on performance metrics for intelligent systems* (ACM), 50–56.

[B17] Mohamed N. Al-Jaroodi J. Jawhar I. (2008). “Middleware for robotics: a survey,” in Ram, 736–742.

[B18] Mohanarajah G. Hunziker D. D’Andrea R. Waibel M. (2014). Rapyuta: a cloud robotics platform. IEEE Trans. Automation Sci. Eng. 12, 481–493. 10.1109/tase.2014.2329556

[B19] Newman S. (2015). Building microservices: designing fine-grained systems. Sebastopol, CA: O’Reilly Media, Inc.

[B20] Pot E. Monceaux J. Gelin R. Maisonnier B. (2009). “Choregraphe: a graphical tool for humanoid robot programming,” in RO-MAN 2009 - the 18th IEEE international symposium on robot and human interactive communication, 46–51.

[B21] Randazzo M. Ruzzenenti A. Natale L. (2018). Yarp-ros inter-operation in a 2d navigation task. Front. Robotics AI 5 (5), 5. 10.3389/frobt.2018.00005 PMC7805980 33500892

[B22] Rodrigues R. A. (2017). An adaptive robotics middleware for a cloud-based bridgeOS. Master’s thesis, Istituto Tecnico Lisboa.

[B23] Seo K.-T. Hwang H.-S. Moon I.-Y. Kwon O.-Y. Kim B.-J. (2014). Performance comparison analysis of linux container and virtual machine for building cloud. Adv. Sci. Technol. Lett. 66, 2. 10.14257/astl.2014.66.25

[B24] Triantafyllou P. Afonso Rodrigues R. Chaikunsaeng S. Almeida D. Deacon G. Konstantinova J. (2021). A methodology for approaching the integration of complex robotics systems: illustration through a bimanual manipulation case study. IEEE Robotics Automation Mag. 28, 88–100. 10.1109/MRA.2021.3064759

[B25] Turnbull L. Samanta B. (2013). “Cloud robotics: formation control of a multi robot system utilizing cloud infrastructure,” in 2013 proceedings of IEEE southeastcon (IEEE), 1–4.

[B26] Vahrenkamp N. Wächter M. Kröhnert M. Welke K. Asfour T. (2015). The robot software framework armarx. it-Information Technol. 57, 99–111. 10.1515/itit-2014-1066

[B27] Wang S. Liu X. Zhao J. Christensen H. I. (2019). “Rorg: service robot software management with linux containers,” in 2019 international conference on robotics and automation (ICRA), 584–590. 10.1109/ICRA.2019.8793764

